# The Power of Rapid Reviews for Bridging the Knowledge-to-Action Gap in Evidence-Based Virtual Health Care

**DOI:** 10.2196/54821

**Published:** 2024-05-22

**Authors:** Megan MacPherson, Sarah Rourke

**Affiliations:** 1 Fraser Health Surrey, BC Canada

**Keywords:** virtual health care, rapid reviews, evidence synthesis, evidence-informed decision-making, knowledge translation

## Abstract

Despite the surge in popularity of virtual health care services as a means of delivering health care through technology, the integration of research evidence into practice remains a challenge. Rapid reviews, a type of time-efficient evidence synthesis, offer a potential solution to bridge the gap between knowledge and action. This paper aims to highlight the experiences of the Fraser Health Authority’s Virtual Health team in conducting rapid reviews. This paper discusses the experiences of the Virtual Health team in conducting 15 rapid reviews over the course of 1.5 years and the benefit of involving diverse stakeholders including researchers, project and clinical leads, and students for the creation of user-friendly knowledge products to summarize results. The Virtual Health team found rapid reviews to be a valuable tool for evidence-informed decision-making in virtual health care. Involving stakeholders and focusing on implementation considerations are crucial for maximizing the impact of rapid reviews. Health care decision makers are encouraged to consider implementing rapid review processes to improve the translation of research evidence into practice, ultimately enhancing patient outcomes and promoting a culture of evidence-informed care.

## Introduction

### Background

Virtual health care services, which involve the delivery of health care through information and communication technologies, have gained popularity among health care providers, patients, and organizations. In recent decades, several initiatives have been undertaken to implement virtual care and improve the access, quality, and safety of health care delivery in Canada [1]; however, technological advancement and a rapidly expanding evidence base make supporting virtual care with research evidence challenging. Specifically, to adequately support virtual care, health care decision makers are expected to keep up with available technologies, their applications, and evidence of their effectiveness among a variety of health conditions.

Despite decision makers recognizing the need to consider research evidence in the context of public health problems [2,3], there is still a knowledge-to-action (KTA) gap between what is known and what is put into practice clinically [4-6], with health care professionals worldwide demonstrating suboptimal use of research evidence within clinical practice [7-14]. Further, it has been estimated that one-third of patients do not receive treatments that have proven efficacious, one-quarter receive treatments that are potentially harmful, and up to three-quarters of patients and half of clinicians do not receive the information necessary for research-informed decision-making [15]. Clearly, there is a need to improve the translation of research evidence into practice, particularly in the case of virtual care where technological innovations and research evidence are rapidly expanding.

### Knowledge Translation

The field of knowledge translation (KT) strives to enhance the usefulness of research evidence through the design and conduct of stakeholder-informed, patient-oriented studies as well as the dissemination and implementation of research findings into practice [16]. The Canadian Institutes for Health Research defines KT as the ethical exchange, synthesis, and application of knowledge among researchers and users to accelerate the benefits of research for Canadian people [17]. The ultimate goal of KT has been further described as the facilitation of evidence-informed decision-making [18] and the integration of various forms of evidence into public health practice and policy.

The Canadian Institutes for Health Research describes 2 “Death Valleys” on the continuum from research to action, which contributes to the KTA gap [19]. Valley 1 refers to the reduced ability to translate basic biomedical research discoveries from the laboratory to the bedside and to effectively commercialize health innovations. Valley 2 refers to the reduced ability to synthesize, disseminate, and integrate research findings more broadly into clinical practice and clinical decision-making. To improve the utility of biomedical and clinical research, enhance health outcomes, and ensure an evidence-based and sustainable health care system, strategic attempts to bridge these valleys must be made.

### Rapid Reviews

One way to help overcome the second valley is through evidence syntheses such as systematic, scoping, and rapid reviews [20]. Evidence syntheses have emerged as valuable methods for KT as they can compile large bodies of evidence into a single knowledge product, making them an essential tool for decision makers to enhance evidence-informed decision-making [21,22]. Systematic reviews offer a comprehensive synthesis of available evidence on a particular topic, playing an ever-expanding role in informing policy making and practice [23,24]; however, the resource-intensive nature of conducting systematic reviews, in terms of both time and cost, presents a significant obstacle to facilitating prompt and efficient decision-making [25].

Given the time constraints health care practitioners and policy makers often face [26], rapid reviews provide a more resource- and time-efficient means to conduct evidence syntheses that offer actionable evidence in a more relevant manner compared to other types of evidence syntheses such as systematic or scoping reviews [20,26-34]. Specifically, rapid reviews are a form of evidence synthesis in which systematic review steps are streamlined to generate actionable evidence within a condensed time frame [35]. To expedite the review process, rapid reviews often compromise on the rigor typically associated with systematic reviews, resulting in a less precise and robust evaluation in comparison [32]. That being said, rapid reviews have gained traction in health systems’ policy making, health-related intervention development, and health technology assessment [34-36]. This paper outlines the experiences of the Fraser Health (FH) Authority Virtual Health team in rapidly producing and disseminating rapid review results to date. Rapid reviews were chosen as they are often highly driven by end-user demands [37] and have been highlighted as a viable tool to disseminate knowledge within the rapidly growing field of virtual health [33].

### FH Authority Context

As the largest regional health authority in British Columbia, Canada, FH serves more than 1.9 million people in Canada [38]. In recent years, FH has prioritized the expansion of virtual care [39], conducting over 1.9 million virtual visits between January 2019 and 2023 (roughly 27% of all visits). Within the Virtual Health department at FH, the “research and evaluation team” aims to improve the translation of research into practice while engaging in ongoing collaborative evaluation of existing Virtual Health programming. During Virtual Health strategic planning, rapid reviews have emerged as a central tool for knowledge dissemination and have been used to inform the development of frameworks, services, and program scale-up. This paper highlights FH’s experience in conducting 15 rapid reviews over the course of 1.5 years. This paper is meant to serve as an overview on the utility and feasibility of rapid reviews within a health authority; for more information on rapid review methods to aid in conducting reviews within a team-based setting, see MacPherson et al [33].

Rapid reviews are used within the Virtual Health team to provide an overview of available evidence addressing a research question related to a single topic produced within a short time frame (typically 1 week to 4 months). From October 2022 until March 2024, the Virtual Health team conducted 15 rapid reviews following published recommendations [33]. Questions posed to date include the following:

1. What are the perspectives on virtual care among immigrant, refugee, and Indigenous people in Canada [40]?
2. What virtual care solutions exist for people with heart failure [41]?
3. What virtual care solutions exist for people with diabetes [41]?
4. What virtual care solutions exist for people with chronic obstructive pulmonary disease (COPD) [41]?
5. What are currently used decision guides or algorithms to inform escalation within remote patient monitoring services for people with heart failure?
6. What barriers, facilitators, and recommendations exist for remote patient monitoring services within the context of respiratory care [42]?
7. What virtual care or digital innovations are used by physicians in acute care [43]?
8. What barriers and facilitators exist for patient-to-provider virtual messaging (eg, SMS text messaging) [44]?
9. What is the existing evidence for centralized remote patient monitoring services [45]?
10. What domains are included within virtual care frameworks targeting appropriateness and safety?
11. What are patient and provider barriers to virtual care [46]?
12. What is the evidence for virtual hospital programs [47]?
13. What KT strategies exist that could be used by the Virtual Health research and evaluation team in their efforts to translate research findings into practice?
14. What is the available evidence on virtual decision-making and clinical judgment?
15. What is the available evidence for, and are there existing validated assessment criteria for nursing assessment frameworks?

Team members assisting with the rapid reviews included researchers, project leads, clinical leads, and students previously unfamiliar with the review process. Knowledge users within the Virtual Health team (eg, clinical leads and clinical directors) were involved throughout the entirety of the review process from developing the research questions to the presentation of research findings in Virtual Health team meetings and the implementation of findings into Virtual Health practice.

Similar to other rapid reviews [20], results were collated and narratively or visually summarized (eg, through infographics) and presented to Virtual Health team members. The final knowledge products were created to offer a high-level overview of the evidence arranged in a user-friendly manner, aiming to provide VH team members with a high-level understanding of the available evidence [41].

## Experiences and Lessons Learned

### Overview

The Virtual Health team’s journey in conducting 15 rapid reviews over the course of 1.5 years has provided valuable insights into the feasibility and utility of rapid reviews within a health authority setting. These lessons learned are from the perspectives of the authors of this paper. MM is the research and KT lead of the Virtual Health department at the FH Authority. Prior to creating the rapid review program within the Virtual Health department, she has prior experience conducting systematic, scoping, and rapid reviews. SR is a clinical nurse specialist within the Virtual Health department at FH. As a system-level leader, SR leverages evidence to informed clinical and service model changes to optimize patient care and outcomes and support strategic priorities. Prior to her involvement in the Virtual Health rapid review program, SR had no previous experience with conducting evidence reviews.

### Importance of Defining a Clear and Actionable Research Question

Throughout this journey, one of the key lessons learned was about the importance of the research question being actionable to ensure that the results of rapid reviews can be readily integrated into practice. Initially, our reviews had broader scopes aimed at informing future Virtual Health service implementations across various populations such as COPD, diabetes, and heart failure. While these reviews were informative, they did not lead to immediate changes in Virtual Health practice and required strategic efforts to disseminate findings and integrate results into practice. Subsequently, we learned that focusing on specific programs or initiatives within the Virtual Health setting yields more actionable results. For instance, a review focused on identifying patient and provider barriers to virtual care was conducted with the explicit purpose of informing the development of a framework to improve video visit uptake among primary care providers. This targeted approach enabled us to directly address the identified barriers through the development of a framework focused on the uptake of safe and appropriate video visits within primary care.

### Benefits and Challenges Involving Knowledge Users

The involvement of knowledge users such as clinical leads and directors in the rapid review process proved to be invaluable. First, they helped focus the scope of reviews by providing insights into the practical needs and priorities within the FH context. For example, the reviews focusing on virtual care solutions for patients with heart failure, COPD, and diabetes were initiated by 1 of the directors within Virtual Health and included an occupational therapist and clinical nurse specialist on the review team. The diverse insights offered by clinician team members helped shape the review questions, search strategy, and analysis, ensuring it addressed the practical needs in delivering virtual care to this specific patient population.

Second, the engagement of nonresearchers, students, and health care professionals in the review process not only enhanced the quality and relevance of the rapid reviews but also provided an opportunity for experiential learning and professional development. By participating in the rapid review process, students and other team members developed essential skills such as critical appraisal, evidence synthesis, and scientific communication. This approach has the potential to bridge the gap between research and practice by building a generation of clinicians who are well versed in evidence-based practice and can effectively translate research findings into clinical decision-making. For example, a team of nursing students participated in a rapid review focused on algorithms for care escalation within remote patient monitoring services for patients with heart failure. While they lacked prior review experience, their fresh perspectives and familiarity with health care practice as it relates to heart failure brought unique insights helping to shape the clinician-oriented KT efforts.

While involving knowledge users throughout the review process offers numerous benefits, it can also extend the time required to complete a review. This is often due to the necessity for these individuals to familiarize themselves with new software while simultaneously mastering the intricacies of conducting reviews and adhering to all associated steps. For instance, several Virtual Health team members have observed that during their initial and subsequent reviews, they encountered difficulties in efficiently navigating the study screening phase. The abundance of potentially relevant literature posed a challenge, with concerns arising about potentially overlooking papers containing valuable insights or “hidden gems.” This underscores the importance of establishing clear eligibility criteria and providing comprehensive training from the outset to ensure reviewers feel empowered to exclude papers confidently, even those that may initially appear intriguing.

### Resources and Staff Time Involved

Readers interested in starting a rapid review program in their own health systems may find it helpful to understand the resources and staff time involved in our process. As the research and KT lead within the Virtual Health team, MM has been responsible for building the rapid review program, training team members, and leading rapid reviews. Her full-time role allows for dedicated focus on these as well as other research and KT-related activities, ensuring the smooth operation of the rapid review process.

Additionally, strong leadership support within the Virtual Health team has been instrumental in fostering a culture of evidence-informed decision-making and facilitating the integration of research evidence into practice. While we do not have a core team with a dedicated full-time equivalent specifically for rapid reviews, a call is put out to the Virtual Health department at the beginning of a review to identify who has the capacity to assist in a review. A testament to the value of these reviews is that VH team members have begun autonomously conducting rapid reviews with the research and KT lead acting as an advisor, not a lead on the reviews. For example, a nurse who was tasked with creating a framework for a virtual nursing assessment requested assistance in running a search for her team to complete a rapid review, to ensure that the resulting framework did not miss any key components seen in the literature.

### Rapid Review Process

**Figure 1:**
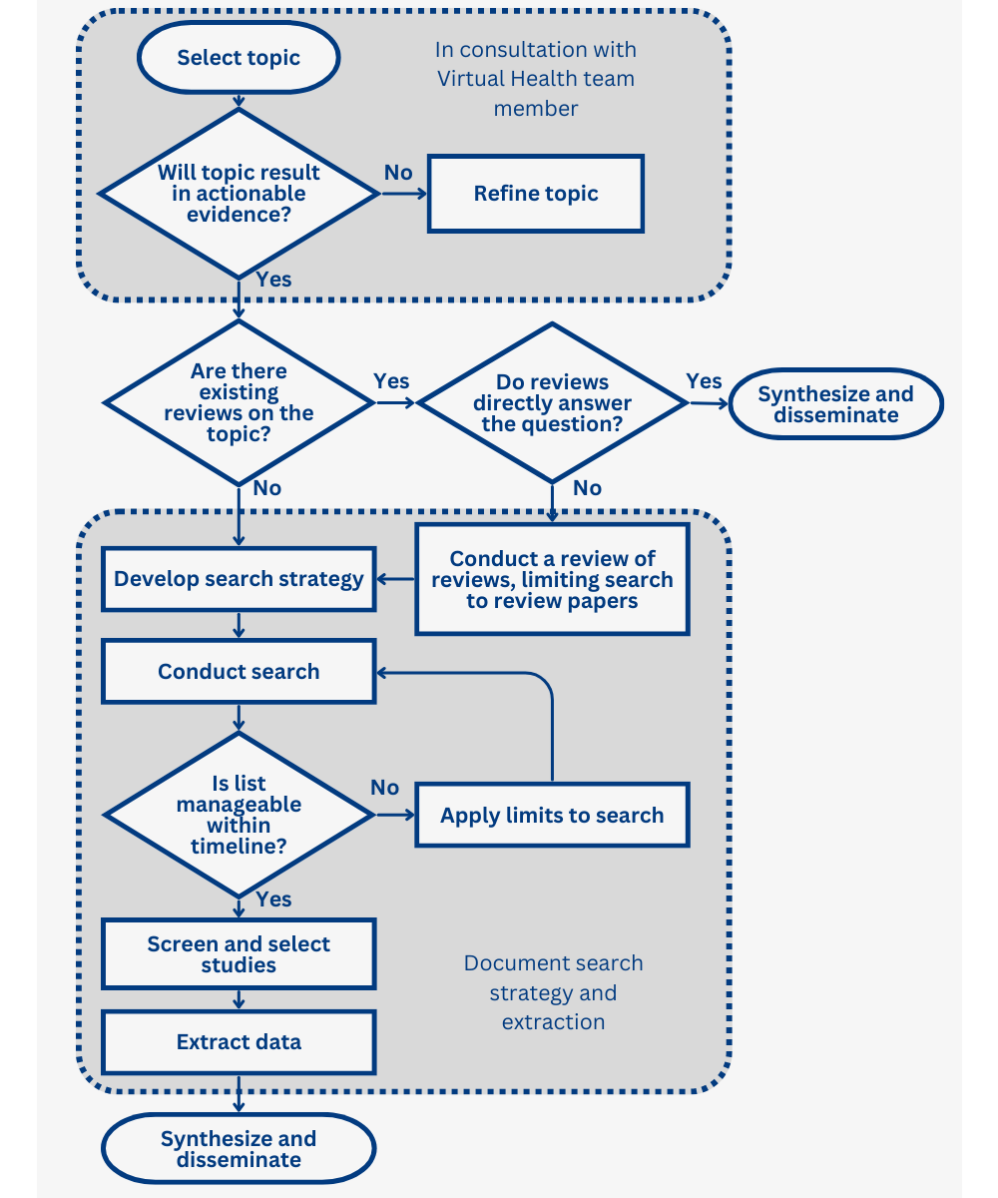
Virtual Health rapid review process map (adapted from MacPherson et al [33], which is published under Creative Commons Attribution 4.0 International License [48]).

The overall process map for our team (an adaptation of MacPherson et al [33,48]) can be found in Figure 1. Our journey in conducting rapid reviews has been accompanied by several challenges and the implementation of quality assurance measures to ensure the integrity of our findings. The overall process of reviews within the Virtual Health team includes Virtual Health team members submitting a request or having an informal meeting with the research and KT lead outlining the scope and purpose of the review, which is then refined to ensure that it will result in actionable evidence relevant to the Virtual Health team and is in alignment with organizational priorities.

Challenges or obstacles encountered during the rapid review process have included resource constraints. When there are not enough people to assist with a review, either the time to complete the review needs to be extended or additional constraints must be placed on the review question. Time limitations have also been a factor, especially when there is an urgent request. Clear communication on how the results will be used is needed to refine the review topic and search strategy to quickly produce actionable evidence. Given the wealth of research, we have started all reviews by first exploring if our questions can be answered by conducting a review of reviews. This has allowed for the timely synthesis of evidence instead of relying on individual studies. We have also found that decision makers value the most up-to-date evidence (especially regarding virtual health care technologies); as such, many of our reviews have imposed limitations to the past 5-10 years to ensure their relevance to decision makers. Additionally, difficulties in accessing relevant literature have been noted, as health authorities often do not have access to the same resources as academic institutions. This results in increased time to secure papers through interlibrary loans, which can be overcome by collaborating with academics.

### Knowledge Translation

Another strength of the Virtual Health team’s rapid review approach was the development of easily digestible knowledge products highlighting key data synthesized in the review. Rather than providing end users with lengthy reports that often go unread, clinicians within the Virtual Health team helped to create brief summaries and infographics highlighting the main findings and recommendations. This approach was aimed at improving the uptake of research evidence into practice by presenting the information in a format that was easily accessible and understandable for clinicians and other stakeholders. By creating visually appealing and user-friendly knowledge products, the Virtual Health team was able to efficiently communicate key takeaways from the rapid reviews, thus facilitating their dissemination and implementation within the FH context. This approach also helped to overcome a common challenge of KT, where research evidence can be difficult to access, understand, and apply in practice. By presenting the information in a format that was relevant and easily digestible, the Virtual Health team was able to enhance the applicability of the rapid reviews, thereby building clinician capacity and increasing their potential impact on patient outcomes.

### Leveraging Rapid Reviews for Clinically Based Tools

Our most recent reviews were focused on developing a virtual nursing assessment and virtual nursing decision-making framework. Unlike traditional KT efforts used within other reviews, where the focus often lies on creating user-friendly summaries and infographics, our approach took a slightly different path. We aimed to directly inform the development of clinical decision support tools (DSTs).

Rather than developing traditional KT products, the raw data extracted from these reviews served as a foundational resource for the development of the clinical DSTs. Each piece of information was carefully referenced and integrated into the tool, providing evidence-based support for specific components and functionalities. This direct integration of research evidence into the tool development process not only strengthened the validity and credibility of the tool but also facilitated the transparent communication of the evidence behind each recommendation or feature.

Within these reviews, the active participation of those who were responsible for the development of the DSTs proved invaluable. Their involvement was crucial in ensuring understanding and confidence in the information as well as in merging research evidence with their own clinical expertise. By involving end users in the review process, we could tailor the outcomes to their specific needs and preferences, ultimately enhancing the relevance and applicability of the extracted evidence. This collaborative approach ensured that the resulting DSTs were not only evidence based but also resonated effectively with the clinical context they were intended for.

## Discussion

### Principal Findings

The Virtual Health team’s experience with conducting 15 rapid reviews over the course of 1.5 years highlights the potential of rapid reviews as a time-efficient tool for improving the translation and uptake of research evidence into Virtual Health programming. Compared to more traditional review types (eg, systematic or scoping), which can take more than a year to complete [49], rapid reviews provide a practical way of synthesizing available evidence to inform clinical decision-making. The ability to produce a high-quality evidence summary in a shorter time frame can be particularly valuable in rapidly evolving areas of health care, such as virtual health. While rapid reviews are not new, our program offers insights into their application in a dynamic and rapidly evolving field such as virtual health. The lessons learned from FH’s rapid review program have important implications for evidence-based decision-making and KT within health care settings.

One of our primary lessons learned underscores the importance of establishing clear and actionable research questions. By outlining precise objectives, rapid reviews can ensure the relevance and applicability of their results, thus facilitating their seamless integration into clinical practice. Moreover, our experiences highlight the transformative impact of involving knowledge users throughout the review process. This collaborative approach not only enhances the quality and relevance of the evidence synthesized but also fosters a culture of evidence-informed decision-making within the organization. This type of early and continued engagement of knowledge users in research endeavors has been increasingly recognized as pivotal for establishing research priorities and enhancing the utility of research findings in real-world health care contexts [50,51]. In line with this, the overarching goal of knowledge-user engagement in health research is to coproduce knowledge that directly addresses the needs of decision makers. By involving knowledge users from the outset, research priorities can be aligned with the practical requirements of health care delivery, thereby increasing the relevance and utility of research outputs [52-54].

### Limitations of Rapid Reviews

Despite its benefits, the rapid review approach is not without limitations. Loss of rigor, as mentioned earlier in this paper, remains a concern. The rapid nature of the process may compromise the depth and comprehensiveness of the literature search and synthesis, potentially leading to oversights or biases in the evidence presented. Furthermore, within the context of virtual health, the rapid pace of technological advancements poses a challenge. New technologies may outpace the generation of peer-reviewed literature, resulting in a lag between their implementation and the availability of robust evidence.

In response to the challenge posed by rapidly evolving technologies, FH’s Virtual Health department has used creative solutions to capture relevant evidence. While peer-reviewed literature remains a primary source, we have also incorporated gray literature, such as news articles, trade publications, and reports, from other health care authorities or departments within the review processes when applicable. Additionally, to supplement reviews and provide more contextual evidence, additional research and evaluation methodologies are used (time permitting) to inform Virtual Health service development such as consulting Patient and Family Advisory Councils within FH, conducting interviews with patient and clinician partners, and conducting analyses on existing data within FH.

### Next Steps for FH’s Rapid Review Program

We remain committed to advancing the rapid review program to meet the evolving needs of the Virtual Health department at FH. While we have heard anecdotally that knowledge users value the user-friendly knowledge products developed for rapid reviews, the next steps of this program include an evaluation of our knowledge dissemination to assess the reach and impact the reviews are having within the Virtual Health department.

### Conclusions

Rapid reviews are a valuable tool for the timely synthesis of available research evidence to inform health care decision-making. The Virtual Health team’s experience with conducting rapid reviews highlights the importance of involving a diverse range of knowledge users in the review process and the need to focus on implementation considerations. By engaging knowledge users beyond designated researchers, and particularly by involving clinicians across the research process, rapid reviews become more robust, applicable, and aligned with the practical needs of health care providers and organizations, which can help to bridge the KTA gap.

## Abbreviations

COPD: chronic obstructive pulmonary disease
DST: decision support tool
FH: Fraser Health
KT: knowledge translation
KTA: knowledge-to-action
